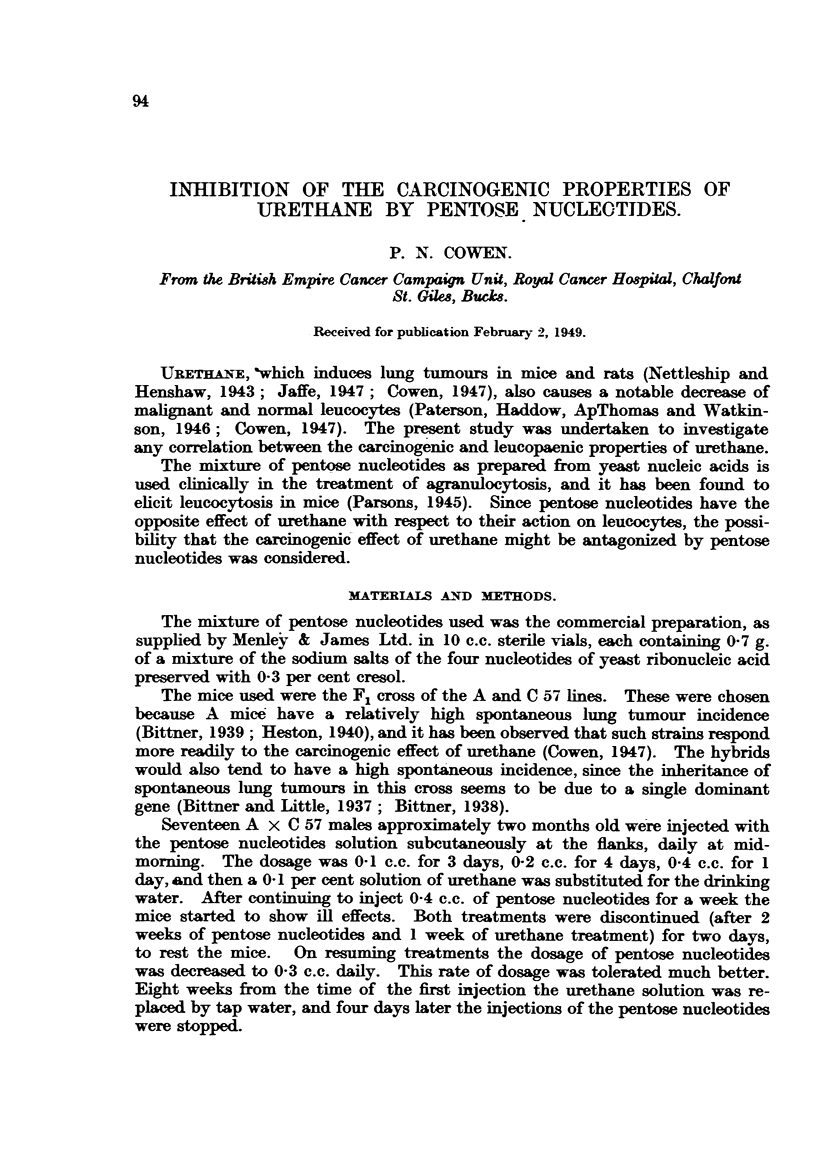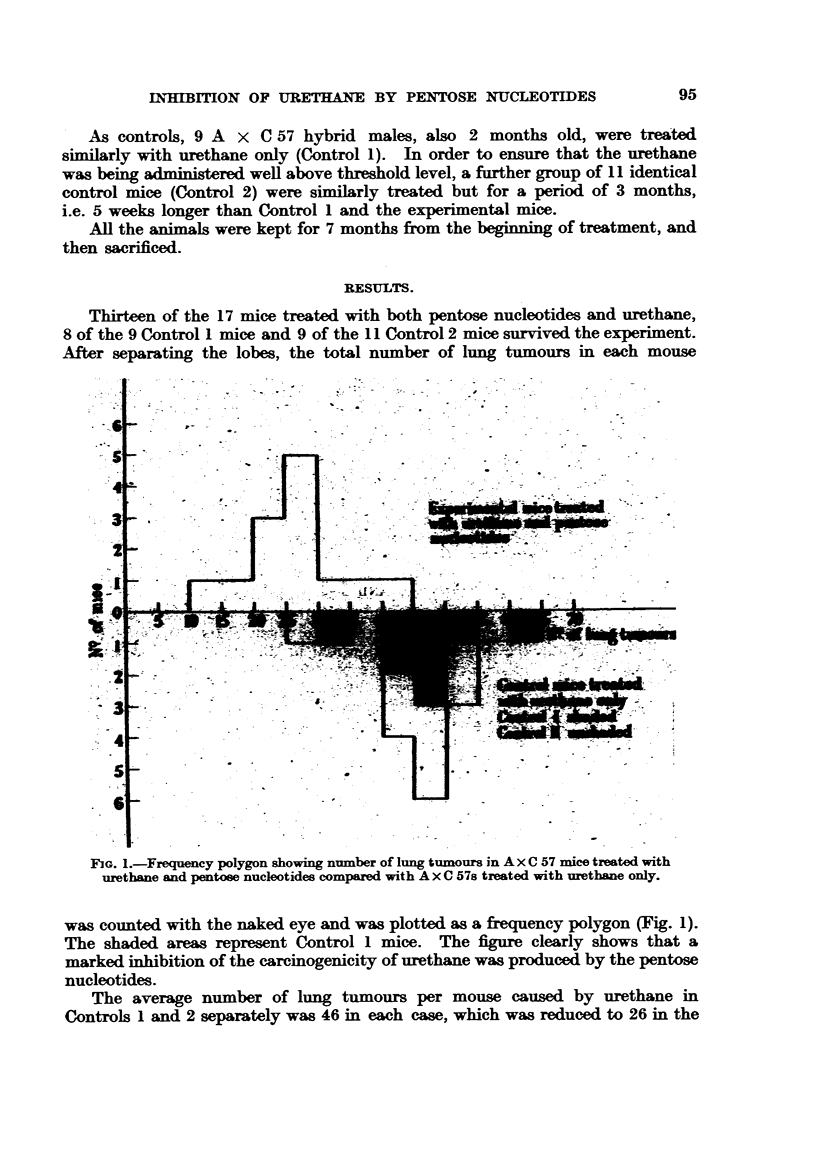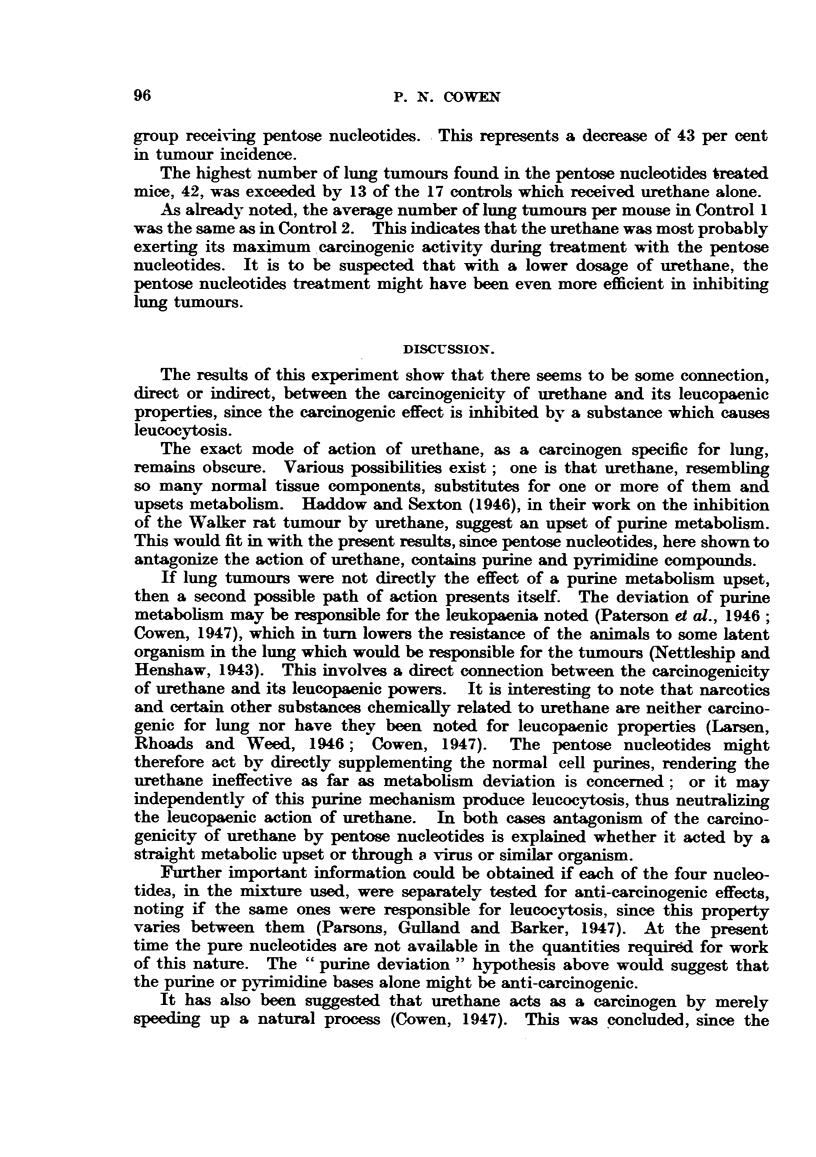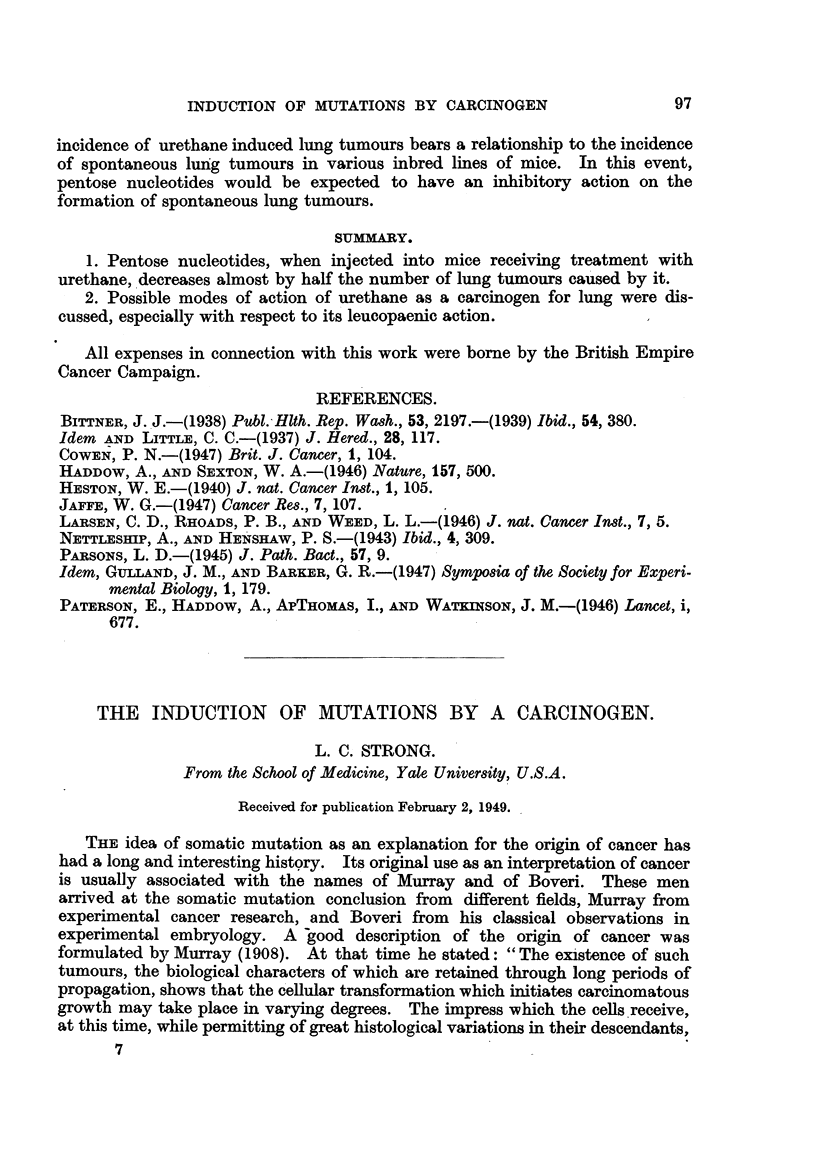# Inhibition of the Carcinogenic Properties of Urethane by Pentose Nucleotides

**DOI:** 10.1038/bjc.1949.11

**Published:** 1949-03

**Authors:** P. N. Cowen


### INHIBITION OF THE CARCINOGENIC PROPERTIES OF URETHANE BY PENTOSE NUCLEOTIDES.

P. N. COWEN.

*From the British Empire Cancer Campaign Unit, Royal Cancer Hospital, Chalfont St. Giles, Bucks.*

Received for publication February 2, 1949.

URETHANE, which induces lung tumours in mice and rats (Nettleship and Henshaw, 1943; Jaffe, 1947; Cowen, 1947), also causes a notable decrease of malignant and normal leucocytes (Paterson, Haddow, ApThomas and Watkinson, 1946; Cowen, 1947). The present study was undertaken to investigate any correlation between the carcinogenic and leucopaenic properties of urethane.

The mixture of pentose nucleotides as prepared from yeast nucleic acids is used clinically in the treatment of agranulocytosis, and it has been found to elicit leucocytosis in mice (Parsons, 1945). Since pentose nucleotides have the opposite effect of urethane with respect to their action on leucocytes, the possibility that the carcinogenic effect of urethane might be antagonized by pentose nucleotides was considered.

### MATERIALS AND METHODS.

The mixture of pentose nucleotides used was the commercial preparation, as supplied by Menley & James Ltd. in 10 c.c. sterile vials, each containing 0·7 g. of a mixture of the sodium salts of the four nucleotides of yeast ribonucleic acid preserved with 0·3 per cent cresol.

The mice used were the $F_1$ cross of the A and C 57 lines. These were chosen because A mice have a relatively high spontaneous lung tumour incidence (Bittner, 1939; Heston, 1940), and it has been observed that such strains respond more readily to the carcinogenic effect of urethane (Cowen, 1947). The hybrids would also tend to have a high spontaneous incidence, since the inheritance of spontaneous lung tumours in this cross seems to be due to a single dominant gene (Bittner and Little, 1937; Bittner, 1938).

Seventeen A $\times$ C 57 males approximately two months old were injected with the pentose nucleotides solution subcutaneously at the flanks, daily at mid-morning. The dosage was 0·1 c.c. for 3 days, 0·2 c.c. for 4 days, 0·4 c.c. for 1 day, and then a 0·1 per cent solution of urethane was substituted for the drinking water. After continuing to inject 0·4 c.c. of pentose nucleotides for a week the mice started to show ill effects. Both treatments were discontinued (after 2 weeks of pentose nucleotides and 1 week of urethane treatment) for two days, to rest the mice. On resuming treatments the dosage of pentose nucleotides was decreased to 0·3 c.c. daily. This rate of dosage was tolerated much better. Eight weeks from the time of the first injection the urethane solution was replaced by tap water, and four days later the injections of the pentose nucleotides were stopped.

As controls, 9 A × C 57 hybrid males, also 2 months old, were treated similarly with urethane only (Control 1). In order to ensure that the urethane was being administered well above threshold level, a further group of 11 identical control mice (Control 2) were similarly treated but for a period of 3 months, i.e. 5 weeks longer than Control 1 and the experimental mice.

All the animals were kept for 7 months from the beginning of treatment, and then sacrificed.

### RESULTS.

Thirteen of the 17 mice treated with both pentose nucleotides and urethane, 8 of the 9 Control 1 mice and 9 of the 11 Control 2 mice survived the experiment. After separating the lobes, the total number of lung tumours in each mouse

FIG. 1.—Frequency polygon showing number of lung tumours in A × C 57 mice treated with urethane and pentose nucleotides compared with A × C 57s treated with urethane only.

was counted with the naked eye and was plotted as a frequency polygon (Fig. 1). The shaded areas represent Control 1 mice. The figure clearly shows that a marked inhibition of the carcinogenicity of urethane was produced by the pentose nucleotides.

The average number of lung tumours per mouse caused by urethane in Controls 1 and 2 separately was 46 in each case, which was reduced to 26 in the group receiving pentose nucleotides. This represents a decrease of 43 per cent in tumour incidence.

The highest number of lung tumours found in the pentose nucleotides treated mice, 42, was exceeded by 13 of the 17 controls which received urethane alone.

As already noted, the average number of lung tumours per mouse in Control 1 was the same as in Control 2. This indicates that the urethane was most probably exerting its maximum carcinogenic activity during treatment with the pentose nucleotides. It is to be suspected that with a lower dosage of urethane, the pentose nucleotides treatment might have been even more efficient in inhibiting lung tumours.

### DISCUSSION.

The results of this experiment show that there seems to be some connection, direct or indirect, between the carcinogenicity of urethane and its leucopaenic properties, since the carcinogenic effect is inhibited by a substance which causes leucocytosis.

The exact mode of action of urethane, as a carcinogen specific for lung, remains obscure. Various possibilities exist ; one is that urethane, resembling so many normal tissue components, substitutes for one or more of them and upsets metabolism. Haddow and Sexton (1946), in their work on the inhibition of the Walker rat tumour by urethane, suggest an upset of purine metabolism. This would fit in with the present results, since pentose nucleotides, here shown to antagonize the action of urethane, contains purine and pyrimidine compounds.

If lung tumours were not directly the effect of a purine metabolism upset, then a second possible path of action presents itself. The deviation of purine metabolism may be responsible for the leukopaenia noted (Paterson *et al.*, 1946 ; Cowen, 1947), which in turn lowers the resistance of the animals to some latent organism in the lung which would be responsible for the tumours (Nettleship and Henshaw, 1943). This involves a direct connection between the carcinogenicity of urethane and its leucopaenic powers. It is interesting to note that narcotics and certain other substances chemically related to urethane are neither carcinogenic for lung nor have they been noted for leucopaenic properties (Larsen, Rhoads and Weed, 1946 ; Cowen, 1947). The pentose nucleotides might therefore act by directly supplementing the normal cell purines, rendering the urethane ineffective as far as metabolism deviation is concerned ; or it may independently of this purine mechanism produce leucocytosis, thus neutralizing the leucopaenic action of urethane. In both cases antagonism of the carcinogenicity of urethane by pentose nucleotides is explained whether it acted by a straight metabolic upset or through a virus or similar organism.

Further important information could be obtained if each of the four nucleotides, in the mixture used, were separately tested for anti-carcinogenic effects, noting if the same ones were responsible for leucocytosis, since this property varies between them (Parsons, Gulland and Barker, 1947). At the present time the pure nucleotides are not available in the quantities required for work of this nature. The " purine deviation " hypothesis above would suggest that the purine or pyrimidine bases alone might be anti-carcinogenic.

It has also been suggested that urethane acts as a carcinogen by merely speeding up a natural process (Cowen, 1947). This was concluded, since the incidence of urethane induced lung tumours bears a relationship to the incidence of spontaneous lung tumours in various inbred lines of mice. In this event, pentose nucleotides would be expected to have an inhibitory action on the formation of spontaneous lung tumours.

SUMMARY.

1. Pentose nucleotides, when injected into mice receiving treatment with urethane, decreases almost by half the number of lung tumours caused by it.

2. Possible modes of action of urethane as a carcinogen for lung were discussed, especially with respect to its leucopaenic action.

All expenses in connection with this work were borne by the British Empire Cancer Campaign.

REFERENCES.

BITTNER, J. J.—(1938) *Publ. Hlth. Rep. Wash.*, **53**, 2197.—(1939) *Ibid.*, **54**, 380.
*Idem* AND LITTLE, C. C.—(1937) *J. Hered.*, **28**, 117.
COWEN, P. N.—(1947) *Brit. J. Cancer*, **1**, 104.
HADDOW, A., AND SEXTON, W. A.—(1946) *Nature*, **157**, 500.
HESTON, W. E.—(1940) *J. nat. Cancer Inst.*, **1**, 105.
JAFFE, W. G.—(1947) *Cancer Res.*, **7**, 107.
LARSEN, C. D., RHOADS, P. B., AND WEED, L. L.—(1946) *J. nat. Cancer Inst.*, **7**, 5.
NETTLESHIP, A., AND HENSHAW, P. S.—(1943) *Ibid.*, **4**, 309.
PARSONS, L. D.—(1945) *J. Path. Bact.*, **57**, 9.
*Idem*, GULLAND, J. M., AND BARKER, G. R.—(1947) *Symposia of the Society for Experimental Biology*, **1**, 179.
PATERSON, E., HADDOW, A., APTHOMAS, I., AND WATKINSON, J. M.—(1946) *Lancet*, i, 677.

---